# Repeated-dose toxicity and immunogenicity evaluation of norovirus P particle-based rotavirus vaccine in juvenile cynomolgus monkeys

**DOI:** 10.3389/fimmu.2026.1841154

**Published:** 2026-06-09

**Authors:** Wei Pan, Yingjie Cheng, Xiaojing Shi, Yunxiang Chen, Cong Xu, Zhengbiao Yang, Qijiong Lu, Lei Wen, Fang Liu, Tingli Bian, Li Qin, Shuizhen Pan, Ying Chen, Changwei Wu, Lijiang Zhang, Hongzhong Yang

**Affiliations:** 1 Key Laboratory of Drug Safety Evaluation and Research of Zhejiang Province, Center of Safety Evaluation and Research, Hangzhou Medical College, Hangzhou, China; 2 Engineering Research Center of Novel Vaccine of Zhejiang Province, Hangzhou Medical College, Hangzhou, China; 3 Anhui Zhifei Longcom Biopharmaceutical Co., Ltd., R&D Department, Hefei, China; 4 Recombinant Vaccine Research and Development Joint Laboratory of Anhui Province, Hefei, China; 5 Zhejiang Key Laboratory of High-level Biosafety and Biomedical Transformation, Proof of Concept Center for Infectious Disease Vaccines and Drugs, Hangzhou Medical College, Hangzhou, China

**Keywords:** bivalent rotavirus vaccine, chimeric virus-like particles, cynomolgus monkeys, immunogenicity, norovirus P particle, repeat-dose toxicity

## Abstract

**Introduction:**

Rotavirus remains a major cause of mortality among young children worldwide, underscoring the urgent need for new vaccines. The present study evaluated the repeated-dose toxicity and immunogenicity of a bivalent rotavirus subunit candidate vaccine PP-P[6]/P[8]-VP8 Mix (containing equal amounts of P[6]-VP8 and P[8]-VP8 chimeric antigens) based on norovirus P particles in cynomolgus monkeys.

**Methods:**

The monkeys (5 per sex per group) were randomly assigned to a blank control group, an adjuvant control group, and low- and high-dose vaccine groups (60 μg/monkey and 120 μg/monkey). The animals received four intramuscular injections at 4-week intervals, followed by a 6-week recovery period. General behavior, body temperature, body weight, food intake, hematology, serum biochemistry, ophthalmic examination, electrocardiogram, urine analysis, immunogenicity parameters, and pathological examination of the animals were detected.

**Results:**

No toxicologically significant abnormalities were observed throughout the study. Histopathological examination showed that there were deposition foci of aluminum hydroxide adjuvant and mild subacute inflammatory reactions at the injection site in the vaccine group. Lymphoid follicle hyperplasia and lymph sinus histiocytosis were observed in the draining lymph nodes, which are typical local immune response manifestations of aluminum adjuvant vaccines. No other adverse pathological changes were found.

**Conclusions:**

Our regulatory toxicology study has demonstrated the safety and immunogenicity of PP-P[6]/P[8] - VP8 Mix under the experimental conditions, supporting its entry into clinical research.

## 1 Introduction

According to data released by the World Health Organization (WHO) in 2024, diarrhea remains the third leading cause of death among children under five years of age globally, accounting for approximately 440,000 child deaths annually (1;Organization, (2024, March 7)). Among these diarrheal cases, rotavirus infection is the primary etiology for acute diarrhea leading to hospitalization and severe gastroenteritis in this age group. In cases of severe rotavirus gastroenteritis (RVGE), frequent diarrhea and vomiting can readily precipitate severe dehydration, electrolyte imbalance, and acid-base disturbances. If not promptly corrected, these physiological derangements may progress to multiple organ failure, and ultimately, death. WHO statistics indicate that RVGE is responsible for up to an estimated 200,000 child deaths each year (2, 3). Due to the absence of specific antiviral agents for rotavirus, the clinical management of RVGE is limited to supportive and symptomatic therapy. Vaccination stands as the most effective measure for preventing RVGE. Since 2009, the WHO has recommended the inclusion of rotavirus vaccines into the National Immunization Program (NIP). Currently, the rotavirus vaccines available for global use are oral live-attenuated vaccines such as Rotarix ^®^, RotaTeq ^®^, Rotasiil ^®^, and Rotavac ^®^, which have demonstrated efficacy in preventing episodes of rotavirus diarrhea. However, vaccine efficacy shows a significant disparity based on country mortality levels (4, 5). Specifically, in low-mortality countries, vaccines confer protection against severe rotavirus diarrhea at rates of 90% to 97%. In contrast, in high-mortality countries, their protective efficacy against severe disease is only 35% to 58% (6). Intussusception, as a rare yet serious adverse event (AE), continues to be a persistent safety concern that requires ongoing monitoring and evaluation for live-attenuated oral vaccines. Consequently, the development of novel vaccines that can efficiently prevent severe rotavirus infection with a superior safety profile represents an urgent public health priority.

Virus-like particles (VLPs) are nanoparticles self-assembled from viral structural proteins, which are highly similar in morphology and structure to their native virus. VLPs exhibit both enhanced immunogenicity and favorable safety profiles. The multimeric structure of VLPs allows for multivalent display of epitopes, which elicits superior cellular and humoral immunity compared to monomeric recombinant protein vaccines. Moreover, due to the absence of viral genetic material, VLPs are non-infectious, circumventing the potential safety risks associated with live-attenuated or inactivated vaccines. To date, several VLP-based vaccines have been approved for clinical use, such as those against hepatitis B, hepatitis E, and human papillomavirus infection (7, 8). Chimeric virus-like particles (cVLPs) are engineered VLPs that serve as platforms for displaying antigen heterologous to the carrier via techniques such as chemical conjugation or genetic fusion. This class of vaccines preserve the high immunogenicity and safety inherent to the VLP platform while providing considerable versatility in antigen selection and combinatorial assembly.

As a prominent example of a chimeric VLP (cVLP), the norovirus P particle serves as an antigen display platform that combines enhanced immunogenicity with structural stability (9, 10). Currently, a range of promising chimeric vaccines based on the P particle platform are currently under development, targeting diseases such as rotavirus(displaying the VP[6], VP[8] epitopes) (11–13),influenza (displaying the M2e epitopes) (14–16), HIV (displaying the 4E10/10E8 epitopes) (17, 18), Alzheimer’s disease (displaying the Aβ1-6) (19, 20). It has been experimentally demonstrated that the norovirus P particle maintains its structural integrity following the insertion peptides of 5–238 amino acids (9). The norovirus P particle is derived from the P domain of the capsid protein VP1, with its S domain removed. It self-assembles into an octahedral structure with a central pore and a diameter of approximately 20 nm. This assembly comprises 12 dimers that form a 24-mer, whose surface displays 24 protruding P domains. The outermost loops of these domains serve as insertion sites for heterologous pathogen antigens, enabling a flexible and multivalent antigen-presentation platform (11). The rotavirus VP8 spike protein is the key functional domain mediating viral attachment to host cells and serves as a primary target for neutralizing antibodies that block viral infection (21, 22). In our previous studies, we successfully constructed a highly immunogenic, multivalent chimeric nanoparticle by efficiently displaying the rotavirus VP8 protein on the protruding domain of the norovirus P particle via genetic fusion (23, 24). To develop a candidate vaccine with broader cross-protectivity, we constructed a bivalent formulation by equally mixing recombinant norovirus P particles separately chimerized with the VP8 antigens from rotavirus genotypes P[6] and P[8]. This candidate vaccine was designated PP-P[6]/P[8]-VP8 Mix.

To date, vaccines based on the norovirus P particle platform still lack systematic preclinical safety data. Consequently, the safety profile of PP-P[6]/P[8]-VP8 Mix—the first bivalent rotavirus candidate vaccine constructed on this platform—remains to be established. Therefore, we conducted a comprehensive preclinical safety assessment of the bivalent formulation composed of PP-P[6]-VP8 and PP-P[8]-VP8, aiming to provide the necessary preclinical safety data to support its first-in-human clinical trial.

## 2 Materials and methods

### 2.1 Construction and preparation of PP-VP8 chimeric vaccine

**Figure 1:**
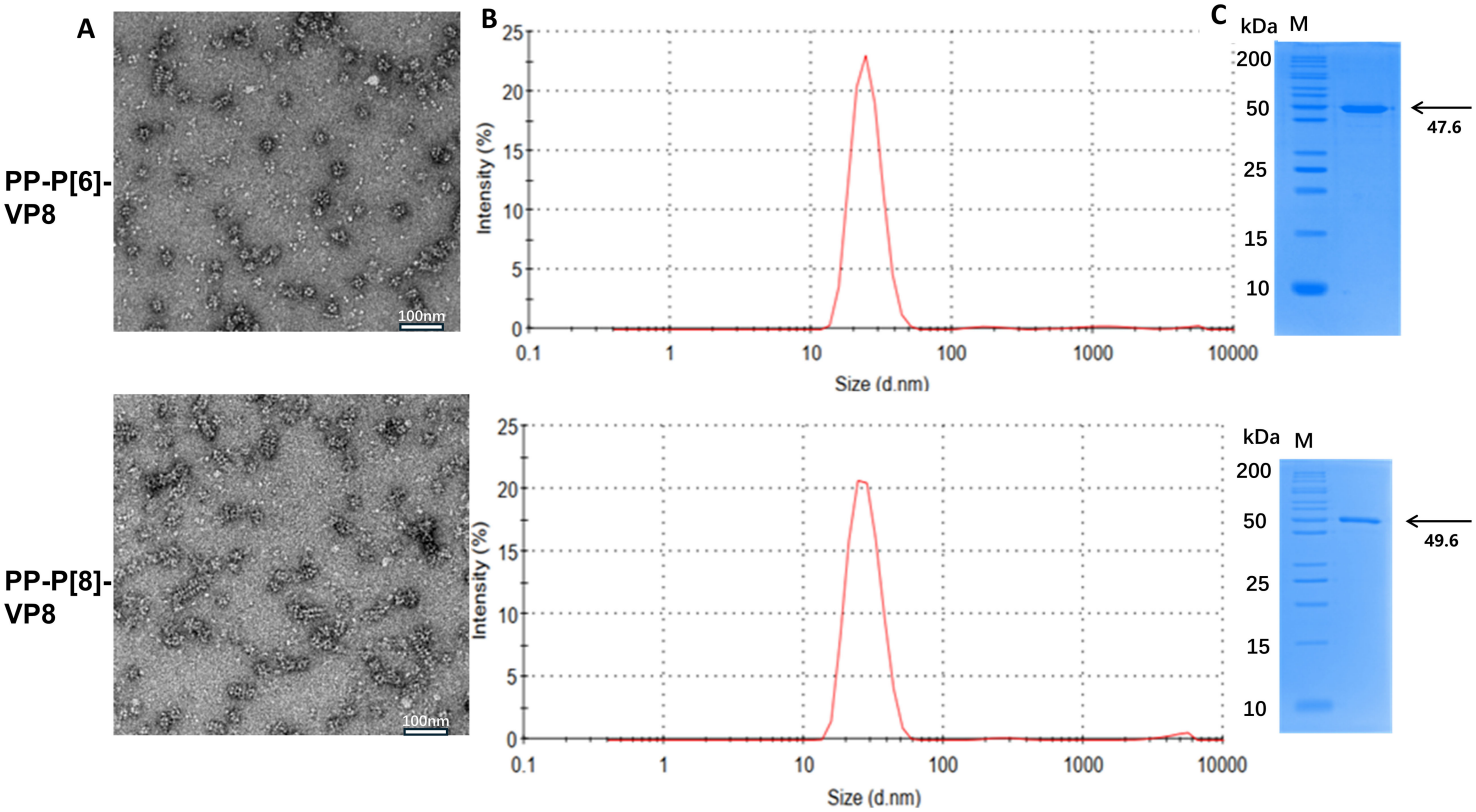
Characterization of particle size, purity, and homogeneity of PP-P[6]-VP8 and PP-P[8]-VP8 monovalent virus-like particles (VLPs). **(A)** Transmission electron microscopy images demonstrate that both PP-P[6]-VP8 and PP-P[8]-VP8 rotavirus multimeric particles are well-assembled, structurally intact, and highly uniform. Scale bar=100 nm. **(B)** Particle size distribution analysis indicates that the diameters of both PP-P[6]-VP8 and PP-P[8]-VP8 particles are approximately 20 nm, with high uniformity. **(C)** Reducing SDS-PAGE analysis shows a single band, corresponding to a molecular mass of 45–55 kDa, indicating high purity of the assembled particles.

We respectively fused the VP8 proteins from rotavirus genotypes P[6] and P[8] to the loop2 motif of the P domain of GII.4 norovirus capsid protein, resulting in the recombinant fusion proteins PP-P[6]-VP8 and PP-P[8]-VP8 (25). Each fusion protein self-assembles into a well-assembled, structurally intact, and highly uniform 24-mer nanoparticle composed of 12 dimers (23, 24)(**Figure 1**). The amino acid sequences of the recombinant fusion proteins PP-VP8 (P[6]-VP8 and P[8]-VP8) were designed based on *Pichia pastoris* codon usage bias and subsequently synthesized as recombinant plasmids by GenScript Biotech (Nanjing, China). The recombinant plasmid was transformed into Pichia Pink™ Strain competent cells (purchased from Thermo Fisher Scientific, USA) to generate a high-yielding recombinant *Pichia pastoris* engineering strain. Subsequently, the establishment of the Master Cell Bank (MCB) and Working Cell Bank (WCB) derived from this engineering strain, along with the fermentation, purification, and final manufacturing of the vaccine product, were all performed by Anhui Zhifei Longcom Biopharmaceutical Co., Ltd. The vaccine, designated PP-P[6]/P[8]-VP8 Mix, was prepared by adsorbing 30 µg each of the PP-P[6]-VP8 and PP-P[8]-VP8 fusion proteins onto aluminum hydroxide in a final volume of 0.5 mL per vial. The corresponding adjuvant control contained an identical concentration of aluminum hydroxide without antigen. The vaccine was stored at 2–8 °C prior to administration.

The vaccine lot met all release specifications: aluminum (0.58 mg/mL), pH (6.26), osmotic pressure (232 mOsm/kg), PEG6000 (10 μg/mL), adsorption (<5% free antigen), endotoxin (<10 EU/mL), and sterility (Chinese Pharmacopoeia).

### 2.2 Animal use and ethical statements

Forty cynomolgus macaques (20 males and 20 females), aged 1.0–1.8 years and weighing between 1.44 and 1.82 kg, were obtained from Guangxi Fangchenggang Biotechnology Development Co., Ltd. (China), holding Certificate of Conformity Number 0003105. Following a quarantine period, all animals were deemed eligible for study inclusion. The animals were housed individually in stainless-steel cages within a facility providing 10–12 filtered air changes per hour, maintained at a temperature of 19–26 °C and 49–69% relative humidity, under a 12:12-h light–dark cycle. A standard 150 g monkey diet was provided daily, supplemented with fresh fruit. Animals were identified using numbered neck tags.

All animal procedures complied with ethical regulations and were approved by the Institutional Animal Care and Use Committee of Hangzhou Medical College Animal Experiments Ethics Committee (Permit Number: GLP-2019-090). The animal facility is accredited by AAALAC International. The study was conducted in accordance with standard operating procedures and Good Laboratory Practice (GLP) guidelines. Euthanasia was performed by intravenous injection of 3% pentobarbital sodium at a dose of 30 mg/kg body weight, followed by exsanguination while the animals were unconscious. The euthanasia procedure conformed to the “AVMA Guidelines for the Euthanasia of Animals, 2013 Edition.

### 2.3 Study design

Following quarantine, animals were randomly assigned to four groups (n = 10) as follows: blank control (Group I), adjuvant control (Group II), low-dose (Group III), and high-dose (Group IV). Groups I and II received 1.0 mL/dose of saline or adjuvant alone, respectively, without P [6]-VP8 or P [8]-VP8 antigens. Groups III and IV received intramuscular injections of the bivalent rotavirus subunit vaccine at doses of 60 μg or 120 μg(1× dose, 2× dose human recommended dose), respectively, into the quadriceps muscle of the hind limbs.

**Figure 2:**
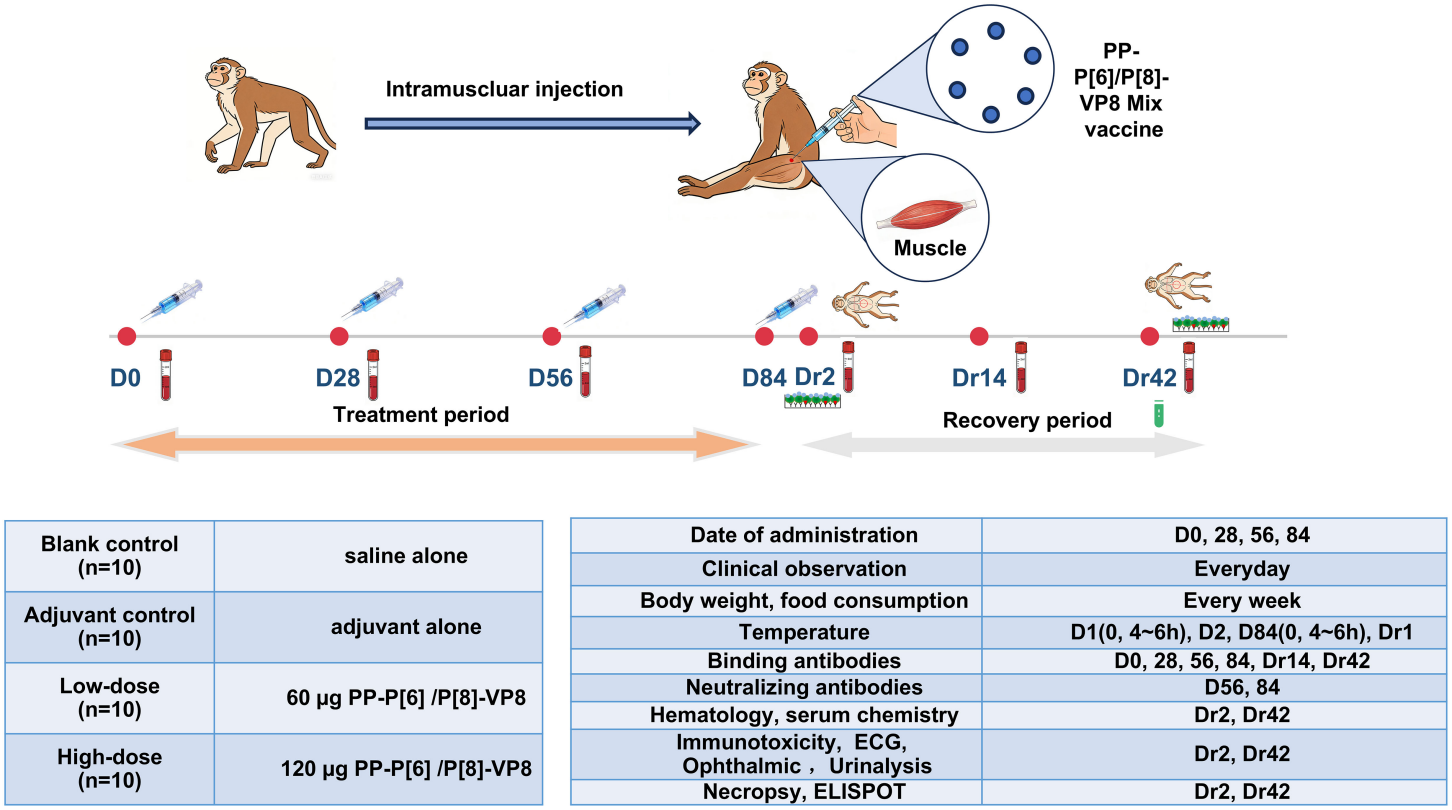
Overview of the repeat-dose toxicity study design for the bivalent subunit rotavirus vaccine. Cynomolgus monkeys received four intramuscular injections of either sodium chloride (blank control), aluminum hydroxide adjuvant (adjuvant control), or the bivalent subunit vaccine (60 μg or 120 μg protein per dose).

In accordance with the National Medical Products Administration guidelines for the preclinical safety evaluation of prophylactic vaccines, monkeys were immunized at baseline (week 0) and then at weeks 4, 8, and 12, for a total of four doses at 4-week intervals—one additional dose beyond the planned clinical regimen of three doses (N + 1 principle). A 6-week recovery phase was included following the final immunization to evaluate the reversibility of any potential vaccine-related toxicities (**Figure 2**).

### 2.4 Clinical examination

Clinical observations were conducted daily, and body weights were recorded weekly throughout the study. Body temperature was measured at baseline, 4–6 hours post-vaccination, and on the day following vaccination at the first (week 0) and final (week 12) doses. Safety assessments, including hematology, blood biochemistry, ophthalmology, urinalysis, ECG, serum immunoglobulins (IgG, IgM), complement components (C3, C4), and peripheral blood lymphocyte subsets (CD3, CD4, CD8), were performed on the second day following the cessation of dosing, designated as Dr2. An additional examination was performed at the end of the recovery period on day 42 (Dr42). At the conclusion of both the dosing and recovery phases (Dr2 and Dr42), six and four animals per group, respectively (equally divided by sex), were euthanized. Major organs—including the heart, liver, spleen, kidneys, brain, thymus, quadriceps muscle, and inguinal lymph nodes—were fixed in 4% paraformaldehyde and paraffin-embedded for histopathological analysis.

### 2.5 ELISA for conjugated antibody titers of vaccines

Serum samples were collected from the adjuvant control, low-dose, and high-dose groups on days 28, 56, and 84 (prior to the second, third, and fourth administrations), as well as on days 14 and 42 following the final administration (Dr14 and Dr42), for the evaluation of immunogenicity. Purified P [6]-VP8 or P [8]-VP8 proteins (Anhui Zhifei Longcom, 20200326, 20200410) were used to coat ELISA plates at a final concentration of 2.5 μg/mL in bicarbonate/carbonate coating buffer and incubated at 37 °C for 2 hours. Following washing with PBST, the plates were blocked with 200 μL of blocking solution (PBST containing 2% [w/v] bovine serum albumin) and incubated overnight at 37 °C. After three additional washes, duplicate wells were filled with 2-fold serial dilutions of serum, starting at 1:2000 (100 μL per well), and incubated for 1 hour at 37 °C. After washing, bound IgG or IgA was detected using horseradish peroxidase-conjugated anti-monkey IgG antibody (Sigma, RI41791) or anti-monkey IgA antibody (Abcam, GR3251324-13), respectively, with incubation at 37 °C for 30 minutes. Following a final set of three washes, 100 μL of 3,3′,5,5′-tetramethylbenzidine substrate (Solarbio, 20191216) was added per well and incubated at room temperature for 10 minutes. The reaction was terminated by adding 50 μL of ELISA stop solution, and the absorbance was measured at 450 nm.

### 2.6 Virus-neutralizing assay

Neutralization assays were conducted using the rapid fluorescent focus inhibition test, in accordance with the guidelines of the Pharmacopoeia of the People’s Republic of China (26;Yager ML, 2015;Rupprecht C.E., 2018;^27^), with minor modifications. Briefly, serum samples were serially diluted two-fold and mixed with an appropriate quantity of Wa or DS-1 rotavirus strain (defined as the amount producing 80% to 95% fluorescent lesion area). MA104 cells, previously seeded in 96-well plates, were infected with 100 μL of serum diluted virus (Wa strain or DS-1 strain) and incubated at 37 °C for 1 hour. After incubation, the cells were washed, and minimum essential medium containing 1 μg/mL trypsin was added. Following an 18-hour incubation, the cells were fixed using 80% acetone at room temperature for 10 minutes. The acetone was discarded, and the plates were air-dried for at least 1 hour. The wells were then washed once with phosphate-buffered saline with Tween-20 (PBST; pH 7.4). A sheep anti-bovine rotavirus polyclonal antibody (Thermo fisher, Cat no. SD2371981A), diluted 1:250, was added at 50 μL per well and incubated overnight at 4 °C. This was followed by the addition of 30 μL per well of fluorescein isothiocyanate -labeled rabbit anti-sheep IgG monoclonal antibody (Sigma, Cat no. 096M4807V), diluted 1: 400, and incubation at 37 °C in a 5% CO_2_ incubator for 1 hour. Fluorescent foci were subsequently enumerated under an inverted fluorescence microscope.

### 2.7 ELISpot detection for cellular immunity

At the end of the dosing phase and the recovery period (Dr2 and Dr42), spleen tissue samples were collected for ELISpot analysis. The frequencies of cells secreting Th1-type cytokines (IL-2, IFN-γ) and the Th2-type cytokine (IL-2, IFN-γ and IL-4 were measured using commercially available ELISpot kits pre-coated with the corresponding capture antibodies, according to the manufacturer’s protocol (MabTech (Cat no. 3445-4APW-2, 3421M-4APT-2), U-CyTech (CT128-PR5)). Briefly, spleens were excised, and spleen lymphocytes were isolated using a monkey spleen lymphocyte separation reagent kit. Each well of the experimental group was stimulated with 100 μL of P [6]-VP8 or P [8]-VP8 at a concentration of 25 μg/mL. Concanavalin A (ConA; 5 μg/mL, Sigma, SLCB0143) and RPMI medium served as positive and negative controls, respectively. A total of 1.5 × 10^6^ spleen lymphocytes were seeded per well and incubated at 37 °C in 5% CO_2_ for 36 hours. Cytokine-secreting cell spots were enumerated using an automated ELISpot reader.

### 2.8 Statistical analysis

Quantitative data—including body weight, growth rate, body temperature, food consumption, hematological and biochemical indices, electrocardiogram parameters, immunological markers, and organ weights and organ-to-body weight ratios—were presented as means ± SD. After confirming the assumptions of homogeneity of variance and normal distribution, group comparisons were performed using one-way analysis of variance (ANOVA) in SPSS software (version 17.0; IBM Corp., Armonk, NY, USA). When the overall difference among groups was significant (P ≤ 0.05), further pairwise comparisons were conducted using the LSD test (for equal variances) or the Dunnett T3 test (for unequal variances). For ordinal variables such as urine parameters, the non-parametric Kruskal–Wallis test was employed. A two-tailed p-value ≤ 0.05 was considered indicative of statistical significance.

## 3 Results

### 3.1 Clinical observations

**Figure 3:**
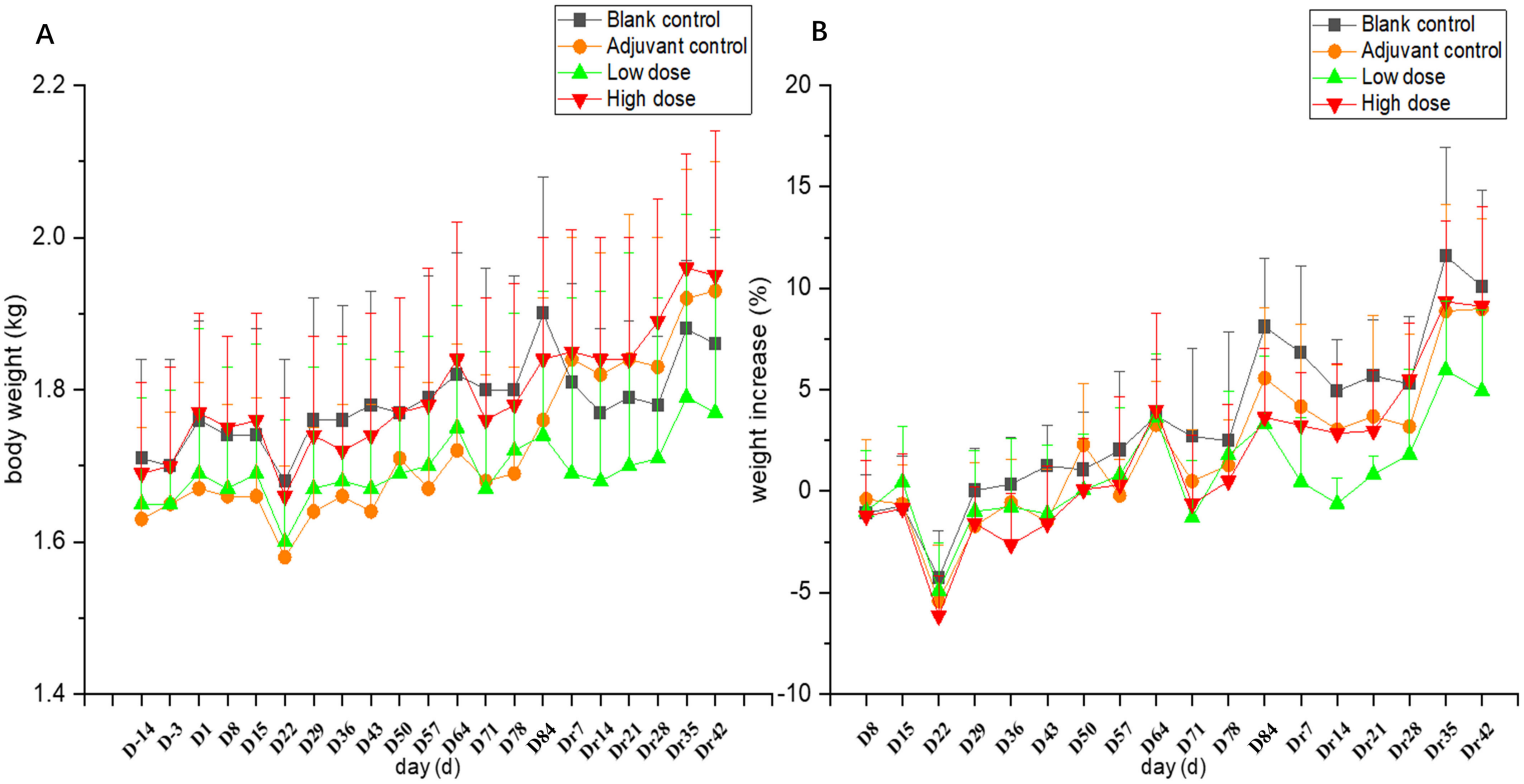
Body weight changes and weight gain **(A, B)** in cynomolgus monkeys following four administrations of the bivalent rotavirus vaccine. n = 10 per group from day −14 to day 84; n = 4 per group from Dr7 to Dr42. Comparisons were made with the control group (*P* > 0.05).

Monkeys received four intramuscular injections of either low- or high-dose vaccine, adjuvant, or saline over a 12-week repeat-dose toxicology study. All animals survived until the end of the experiment. No abnormal clinical symptoms were observed in any group. Body weight exhibited an overall increasing trend throughout the study, with no significant differences among the groups (**Figure 3**). Injection site observations revealed no visible signs of erythema, congestion, swelling, or ulceration.

Body temperature was measured before administration, 4–6 hours after administration, and on the day following both the first and last vaccinations. At 4–6 hours post-initial administration, animals in both the low- and high-dose groups exhibited slightly lower body temperatures compared to the blank control group. Additionally, the high-dose group showed a marginal decrease in body temperature relative to the adjuvant control group (*P* < 0.05, *P* < 0.01). However, mean body temperatures remained within the normal range observed during the adaptation period (**Supplementary Table 1**). These results suggest that the vaccine did not significantly affect body temperature in cynomolgus monkeys.

Food intake was measured for all monkeys throughout the study period, with a total of 20 measurements. Compared to the blank control group, male monkeys in the adjuvant control group exhibited significantly lower food consumption on Days −5 and 81. Conversely, male monkeys in the low-dose group showed significantly higher food intake on Day 39, while those in the high-dose group exhibited reduced consumption on Day −5 and elevated intake on Day 39. Additionally, on Day 81, food intake in both the low- and high-dose male groups was slightly higher than that of the adjuvant control group (*P* < 0.05, *P* < 0.01). No statistically significant differences in food consumption were observed among female monkeys across all groups (*P* > 0.05). Overall, the observed fluctuations in food intake among male monkeys were considered to be toxicologically insignificant (**Supplementary Table 2**).

### 3.2 Hematological and serum biochemical analysis

At the end of the dosing phase, the high-dose group exhibited a higher basophil count (#BASO) compared to the blank control group. Additionally, the low-dose group exhibited elevated hematocrit (HCT) and mean corpuscular volume (MCV) compared to the adjuvant control group. Furthermore, white blood cell count (WBC), monocyte count (#MONO), and fibrinogen (Fbg) levels in both the low- and high-dose groups, as well as #BASO in the low-dose group, were significantly lower than those in the adjuvant control group (*P* < 0.05, *P* < 0.01). There was no significant difference in the hematology during the recovery period. However, these changes remained within both the historical reference range and the physiological variability observed during the acclimatization phase. Therefore, these sporadic statistically significant differences were considered unrelated to vaccine administration. It was concluded that the vaccine did not exert a substantial impact on hematological parameters (**Table 1**).

**Table 1:** Hematology analysis in cynomolgus macaques immunized with the bivalent rotavirus vaccine.

Parameter	End of the dosing phase (n = 10)				End of the recovery phase (n = 4)			
	Blank control	Adjuvant control	Low dose	High dose	Blank control	Adjuvant control	Low dose	High dose
WBC (10e3/μL)	11.27 ± 3.60	19.50 ± 5.36**	12.68 ± 4.13▲	13.87 ± 3.38▲	12.4 ± 4.75	12.96 ± 1.95	13.94 ± 3.31	12.27 ± 3.58
RBC (10e6/μL)	5.52 ± 0.41	5.47 ± 0.33	5.47 ± 0.31	5.55 ± 0.28	6.22 ± 0.61	6.02 ± 0.27	5.79 ± 0.22	5.83 ± 0.31
HGB (g/dL)	133 ± 5	127 ± 6	133 ± 5	131 ± 8	152 ± 5	148 ± 6	142 ± 3	144 ± 7
HCT (%)	43.2 ± 1.9	41.2 ± 2.2*	44.1 ± 2.2▲	43.1 ± 2.1	47.7 ± 1.6	45.5 ± 2.0	44.6 ± 0.5	45.5 ± 2
MCV (fL)	78.4 ± 4.6	75.5 ± 3.2	80.7 ± 4.4▲	77.7 ± 2.0	77.0 ± 4.9	75.8 ± 2.5	77.2 ± 3.1	78.1 ± 2.7
MCH (pg)	24.2 ± 1.6	23.2 ± 0.8	24.3 ± 1	23.6 ± 0.7	24.6 ± 1.8	24.6 ± 0.9	24.6 ± 1.1	24.6 ± 0.4
MCHC (g/dL)	309 ± 7	308 ± 7	301 ± 7	304 ± 8	319 ± 5	324 ± 8	319 ± 4	316 ± 10
RDW (%)	12.7 ± 0.6	13.1 ± 0.6	12.9 ± 0.8	13.1 ± 0.7	12.1 ± 0.3	12.3 ± 0.5	12.2 ± 0.6	12.7 ± 0.7
PLT (10e3/μL)	371 ± 62	345 ± 71	432 ± 111	421 ± 70	394 ± 75	358 ± 67	504 ± 138	431 ± 120
MPV (fL)	8.9 ± 0.9	8.8 ± 0.9	8.9 ± 0.7	8.8 ± 1.2	8.7 ± 0.4	9.0 ± 1.1	8.2 ± 1.2	8.1 ± 0.6
#NEUT (10e3/μL)	3.73 ± 1.63	11.02 ± 5.56*	5.5 ± 3.3	5.47 ± 2.01	4.36 ± 2.00	3.53 ± 0.80	5.68 ± 4.48	6.85 ± 1.85
#LYMPH (10e3/μL)	6.54 ± 2.29	6.88 ± 2.09	6.16 ± 2.21	7.03 ± 2.69	6.94 ± 2.25	8.41 ± 1.33	7.39 ± 2.70	4.38 ± 1.35
#MONO (10e3/μL)	0.44 ± 0.21	0.97 ± 0.40**	0.53 ± 0.25▲	0.66 ± 0.29△	0.46 ± 0.26	0.49 ± 0.26	0.39 ± 0.05	0.49 ± 0.17
#EOS (10e3/μL)	0.39 ± 0.16	0.37 ± 0.31	0.33 ± 0.22	0.49 ± 0.39	0.42 ± 0.22	0.22 ± 0.18	0.26 ± 0.13	0.40 ± 0.29
#BASO (10e3/μL)	0.05 ± 0.03	0.09 ± 0.03*	0.05 ± 0.02△	0.08 ± 0.05*	0.08 ± 0.04	0.08 ± 0.01	0.06 ± 0.03	0.06 ± 0.02
#LUC (10e3/μL)	0.12 ± 0.05	0.19 ± 0.10	0.12 ± 0.04	0.14 ± 0.08	0.14 ± 0.06	0.23 ± 0.08	0.15 ± 0.08	0.10 ± 0.03
#RETIC (10e9/L)	60.0 ± 14.0	56.3 ± 13.1	62.0 ± 18.5	68.4 ± 18.6	59.6 ± 24.6	55.7 ± 6.2	46.2 ± 12.0	54.9 ± 13.1
PT (s)	12.2 ± 2.6	11.1 ± 0.5	11.6 ± 1.6	10.7 ± 0.8	11.6 ± 1.6	10.8 ± 0.8	10.9 ± 0.4	11.8 ± 0.7
Fbg (g/L)	2.012 ± 0.263	2.469 ± 0.563**	2.129 ± 0.206△	1.975 ± 0.282▲	1.784 ± 0.332	1.869 ± 0.365	1.974 ± 0.145	1.830 ± 0.201
APTT (s)	29.4 ± 6.3	27 ± 3.1	27.1 ± 1.9	24.8 ± 1.2	27.8 ± 5.5	28.4 ± 2.7	25.3 ± 0.4	25.3 ± 1.2

Compared to the saline control group * *P* < 0.05 and ** *P* < 0.01. Compared to the adjuvant-only group ▲*P* < 0.05 and △*P* < 0.01.

At the end of the dosing phase, creatinine levels in the vaccine-treated groups were higher than those in the adjuvant control group. Additionally, a statistically significant difference was observed between the vaccine-treated and blank control groups for two key electrolytes: sodium (Na^+^) and calcium (Ca²^+^). Nevertheless, these minor alterations remained within the normal fluctuation range recorded during the adaptation period and were considered to lack toxicological significance (**Table 2**).

**Table 2:** Blood biochemistry analysis in cynomolgus macaques immunized with the bivalent rotavirus vaccine.

Parameter	End of the dosing phase (n = 10)				End of the recovery phase (n = 4)			
	Blank control	Adjuvant control	Low dose	High dose	Blank control	Adjuvant control	Low dose	High dose
ALT (IU/L)	47.33 ± 11.91	52.90 ± 12.97	50.75 ± 18.04	54.03 ± 14.66	45.65 ± 3.06	49.39 ± 9.08	48.45 ± 14.17	36.27 ± 6.18
AST (IU/L)	56.51 ± 11.79	54.36 ± 9.54	59.91 ± 7.85	58.43 ± 11.42	54.80 ± 6.65	51.62 ± 6.38	61.50 ± 19.13	47.62 ± 5.96
T.P (g/L)	79.96 ± 4.31	80.08 ± 4.84	82.58 ± 4.20	83.18 ± 3.01	73.34 ± 4.11	74.54 ± 6.26	72.56 ± 3.56	69.71 ± 3.69
ALB (g/L)	41.98 ± 1.74	40.84 ± 1.54	42.53 ± 1.75	41.51 ± 1.42	43.69 ± 1.15	44.21 ± 2.93	42.20 ± 1.83	42.31 ± 2.35
T.BIL (μmol/L)	2.911 ± 0.691	2.383 ± 0.494	3.088 ± 1.134	2.737 ± 0.587	3.720 ± 1.015	3.038 ± 0.514	3.189 ± 1.627	3.54 ± 0.662
ALP (IU/L)	626.5 ± 214.47	628.59 ± 197.17	592.59 ± 73.43	597.38 ± 162.92	743.81 ± 346.43	583.47 ± 102.88	571.60 ± 258.78	643.16 ± 149.73
GGT (IU/L)	90.67 ± 24.06	70.17 ± 17.87	71.51 ± 20.26	91.24 ± 27.37	108.40 ± 32.30	91.66 ± 10.40	75.82 ± 14.31	102.11 ± 37.72
GLU (mmol/L)	3.679 ± 0.878	3.948 ± 0.973	3.535 ± 1.112	4.096 ± 0.717	3.012 ± 0.336	3.237 ± 0.758	3.050 ± 0.711	3.203 ± 0.345
BUN (mmol/L)	7.486 ± 1.286	6.644 ± 1.073	7.693 ± 1.373	6.718 ± 0.836	9.361 ± 1.126	8.017 ± 1.633	8.794 ± 1.888	8.620 ± 1.485
Crea (μmol/L)	56.11 ± 4.73	52.22 ± 6.14	58.77 ± 7.13△	59.04 ± 4.81△	64.85 ± 7.03	52.88 ± 1.91**	57.07 ± 5.95	60.78 ± 4.46
T.CHO (mmol/L)	3.179 ± 0.760	3.150 ± 0.451	3.463 ± 0.57	3.338 ± 0.669	3.796 ± 0.728	3.915 ± 0.283	3.566 ± 0.693	3.252 ± 0.628
TG (mmol/L)	0.240 ± 0.087	0.262 ± 0.080	0.291 ± 0.090	0.302 ± 0.093	0.424 ± 0.080	0.317 ± 0.119	0.318 ± 0.066	0.431 ± 0.079
CK (IU/L)	291.5 ± 128.1	309.3 ± 181.3	273.6 ± 76.6	316.2 ± 84.9	309.2 ± 58.6	334.8 ± 67.6	365.0 ± 80.8	349.8 ± 156.2
GLO (g/L)	37.98 ± 2.99	39.24 ± 3.84	40.05 ± 4.27	41.67 ± 2.63	29.65 ± 3.29	30.33 ± 3.57	30.36 ± 3.44	27.41 ± 3.33
A/G	1.11 ± 0.07	1.05 ± 0.09	1.07 ± 0.14	1.00 ± 0.07	1.48 ± 0.14	1.47 ± 0.10	1.41 ± 0.17	1.56 ± 0.21
K^+^ (mmol/L)	3.93 ± 0.52	3.66 ± 0.41	3.88 ± 0.50	3.92 ± 0.21	4.19 ± 0.37	3.96 ± 0.30	3.93 ± 0.32	4.13 ± 0.44
Na^+^ (mmol/L)	149 ± 1	151 ± 2*	152 ± 2**	152 ± 2**	148 ± 1	147 ± 1	149 ± 2	150 ± 2
Cl^-^ (mmol/L)	110.0 ± 1.9	110.9 ± 2.1	110.2 ± 2.2	109.8 ± 1.8	104.3 ± 2.1	104.1 ± 1	105.5 ± 0.8	105.7 ± 1.9
Ca (mmol/L)	2.56 ± 0.08	2.48 ± 0.05**	2.52 ± 0.06	2.47 ± 0.08**	2.68 ± 0.08	2.65 ± 0.06	2.65 ± 0.10	2.60 ± 0.06

Compared to the saline control group, * *P* < 0.05 and ** *P* < 0.01. Compared to the adjuvant-only group, △*P* < 0.01.

### 3.3 ECG, immunotoxicology, ophthalmology and urinalysis examinations

At the end of the dosing phase, the P wave amplitude was lower in the low-dose group than that in the blank control group. The heart rate in the high-dose group was reduced compared to both the blank and adjuvant control groups. Following the recovery phase, the R wave amplitude in the low-dose group increased above the levels observed in the blank and adjuvant control groups (**Supplementary Table 3**). As all electrocardiograms (ECGs) maintained normal sinus rhythm and the noted changes remained within the scope of physiological variability observed during acclimatization, they were therefore regarded as incidental fluctuations without toxicological relevance.

Peripheral blood immunoglobulins (IgG, IgM), complement proteins C3 and C4, and T−cell subset percentages (CD3^+^, CD3^+^CD4^+^, CD3^+^CD8^+^ cells, along with the CD4^+^/CD8^+^ ratio) were assessed at the end of the both dosing and recovery phases, revealed no significant inter-group difference (**Table 3**).

**Table 3:** Immunotoxicological indices in cynomolgus macaques immunized with the bivalent rotavirus vaccine.

Parameter	End of the dosing phase (n = 10)				End of the recovery phase (n = 4)			
	Blank control	Adjuvant control	Low dose	High dose	Blank control	Adjuvant control	Low dose	High dose
IgG (g/L)	9.00 ± 1.12	9.56 ± 2.29	9.72 ± 2.17	10.84 ± 2.17	9.83 ± 2.34	10.97 ± 1.85	11.50 ± 2.23	11.28 ± 3.13
IgM (g/L)	0.39 ± 0.16	0.50 ± 0.19	0.44 ± 0.18	0.46 ± 0.20	0.81 ± 0.30	0.86 ± 0.34	0.77 ± 0.22	0.49 ± 0.26
C3 (mg/dL)	104.74 ± 15.95	103.43 ± 20.98	110.66 ± 23.30	107.01 ± 16.73	102.90 ± 21.33	114.26 ± 32.76	114.60 ± 16.19	107.97 ± 22.19
C4 (mg/dL)	22.73 ± 6.98	28.87 ± 9.75	30.50 ± 8.33	29.70 ± 8.03	18.35 ± 8.33	24.99 ± 8.38	28.67 ± 6.06	34.82 ± 7.72
CD3^+^ (%)	56.78 ± 10.65	48.74 ± 8.13	55.68 ± 9.26	53.58 ± 6.15	51.02 ± 10.51	59.43 ± 5.39	51.87 ± 7.26	43.95 ± 3.95
CD3^+^CD4^+^ (%)	55.75 ± 7.01	50.92 ± 8.52	52.29 ± 6.69	49.65 ± 7.54	52.34 ± 7.06	48.47 ± 6.48	49.22 ± 8.93	55.11 ± 5.49
CD3^+^CD8^+^ (%)	35.81 ± 6.77	40.62 ± 9.60	38.51 ± 4.70	40.98 ± 8.88	37.14 ± 6.75	43.14 ± 5.99	39.84 ± 5.74	32.91 ± 7.66
CD4^+^/CD8^+^	1.64 ± 0.50	1.38 ± 0.59	1.39 ± 0.29	1.29 ± 0.40	1.47 ± 0.48	1.15 ± 0.31	1.28 ± 0.42	1.79 ± 0.64

No statistically significant differences between the groups (*P* > 0.05).

Ophthalmologic and urinalysis examinations revealed no abnormalities in any of the animals.

### 3.4 Binding and neutralizing antibody titers

**Figure 4:**
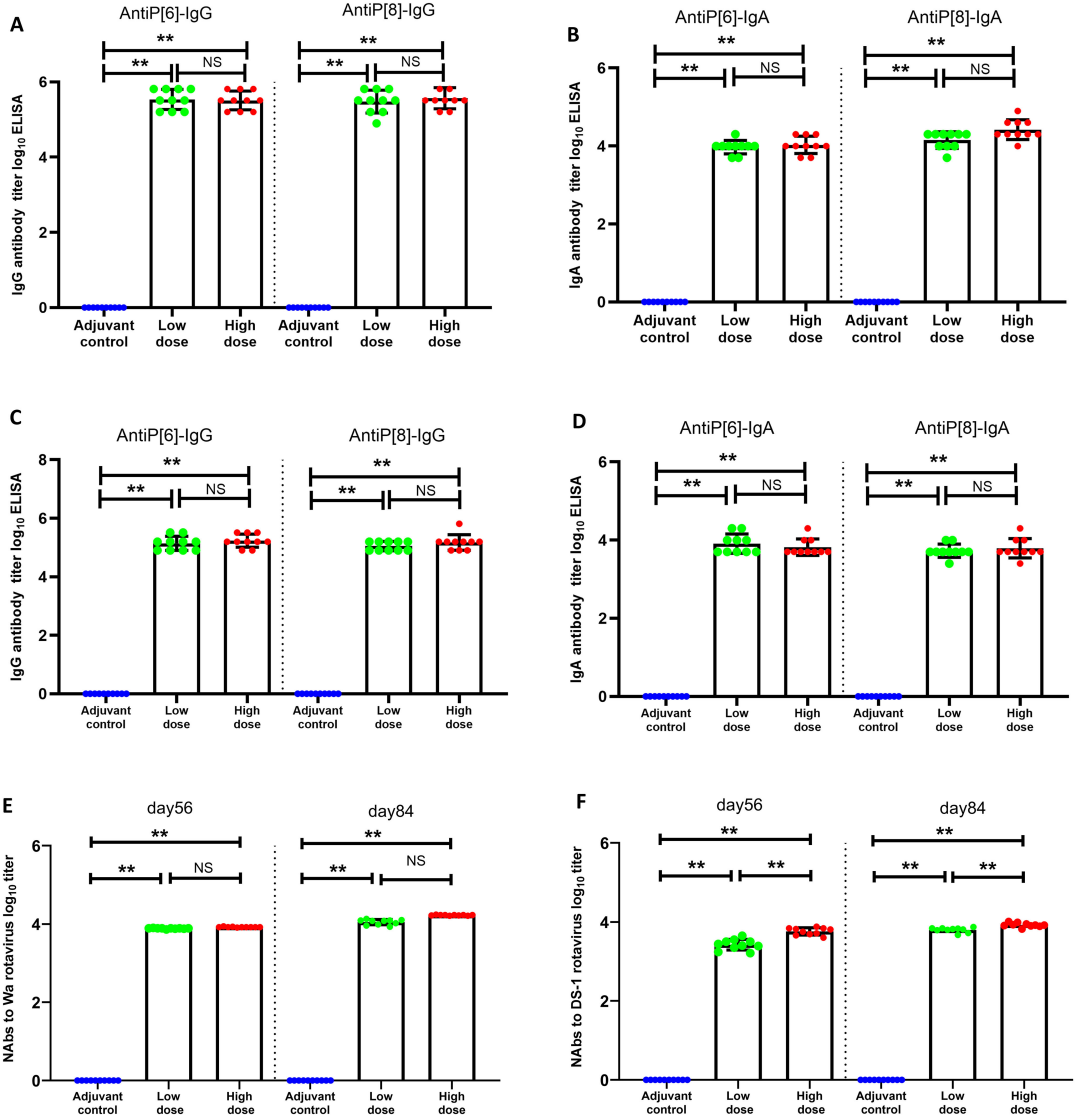
Humoral immune response to the bivalent rotavirus vaccine in cynomolgus monkeys. **(A, B)** Serum IgG and IgA binding antibodies specific to P[6]-VP8 and P[8]-VP8 on day 57 were quantified using indirect ELISA. **(C, D)** Serum IgG and IgA binding antibodies specific to P[6]-VP8 and P[8]-VP8 on day 57 were quantified using indirect ELISA. **(E, F)** Neutralizing antibody titers against the Wa (G1P[8]), DS-1(G2P[4]) strains were measured in serum samples collected on days 56 and 84 using a virus neutralization assay. n = 10 animals/group. Comparisons were made using one-way analysis of variance (ANOVA). **P < 0.01 compared to the adjuvant control group.

Following three intramuscular injections of the bivalent rotavirus vaccine at doses of 60 μg (low-dose group) or 120 μg (high-dose group) per animal on Day 56, serum samples were collected and analyzed to determine IgG and IgA binding antibody levels by enzyme-linked immunosorbent assay (ELISA). Both low- and high-dose groups produced P[6]-VP8- and P[8]-VP8-specific IgG and immunoglobulin A (IgA) binding antibodies. The binding antibody positivity rate reached 100% in both dosage groups. Titers of IgG and IgA binding antibodies against P[6]-VP8 and P[8]-VP8 were significantly higher in both low- and high-dose groups compared to the adjuvant control group (*P* < 0.01). No statistically significant difference in antibody titers was observed between the low- and high-dose groups. The increase in serum IgG antibodies was coincided with those of IgA antibodies response (**Figures 4A, B**). Serum IgG and IgA antibody levels measured on Day 84 exhibited a similar pattern of change following vaccine administration as those observed on Day 56. (**Figures 4C, D**).

To assess the neutralizing activity induced by the bivalent vaccine, blood samples were analyzed on Days 56 and 84. Both low- and high-dose of the bivalent rotavirus vaccine successfully induced neutralizing antibodies against the Wa strain (G1P[8]) in macaques, with a neutralizing antibody positivity rate of 100%. The neutralizing antibody titers against serotype P[8] rotavirus exceeded 3.9 log_10_ TCID_50_ on day 56 and remained elevated through the end of the dosing period of day 84 (**Figure 4E**). Due to the unavailability of a P[6] virus strain during the course of this study, neutralization assays against a representative P[6] strain were not performed. To preliminarily evaluate the vaccine’s potential cross- neutralization capacity, we instead examined the neutralizing activity of immunized serum against the heterologous DS-1 type strain (G2P[4]). Both the low- and high-dose groups of macaque monkeys exhibited significantly higher levels of neutralizing antibodies against DS-1 strain (serotype P[4]) rotavirus after two and three doses (Day56, Day84), and the neutralizing antibody positivity rate was 100%, with the high-dose group showing notably higher titers compared to the low-dose group (*P* < 0.01) (**Figure 4F**). The bivalent vaccine-elicited sera showed potent neutralization against the homologous P[8] strain (Wa) and the heterologous P[4] strain (DS-1), indicating its potential to induce cross-protective immunity.

### 3.5 Kinetics of P[6]- and P[8]-specific IgG antibodies

**Figure 5:**
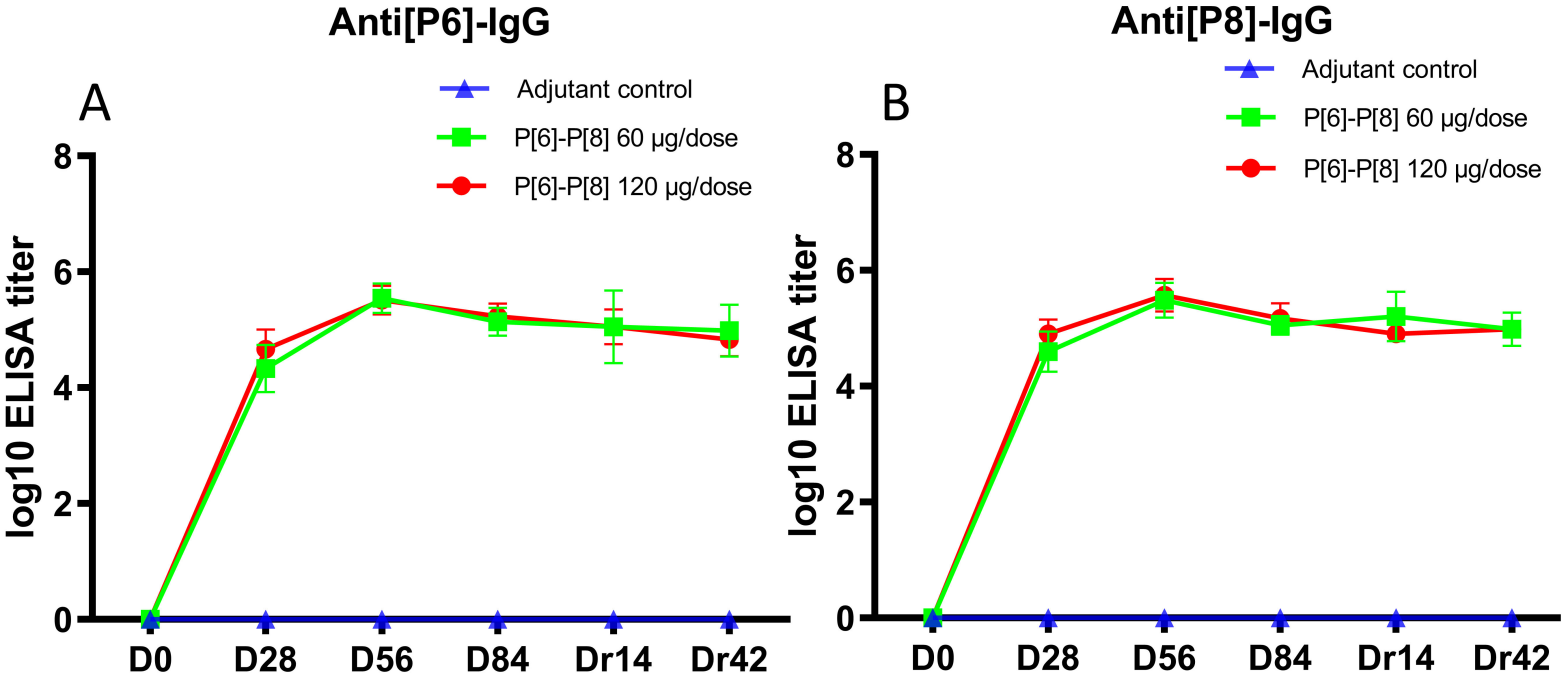
Kinetics of serum-specific IgG antibodies. Sample size: n = 10 animals/group on days 28, 56, and 84; n = 4 animals/group on Dr14 and Dr42. Results are expressed as mean ± SD. **(A)** Serum IgG levels against P[6]-VP8. **(B)** Serum IgG levels against P[8]-VP8.

To evaluate the time-course of specific immunoglobulin G (IgG) responses against P[6]-VP8 and P[8]-VP8 in cynomolgus monkeys, antibody levels were measured at six time points: Day 0, Day 28, Day 56, Day 84, Dr14, and Dr42. After the first vaccination (Day 28), high-titer antibodies against P[6]-VP8 were produced, with geometric mean titers (GMTs) of log_10_(4.33) in the low-dose group and log_10_(4.66) in the high-dose group. Similarly, high-titer antibodies against P[8]-VP8 were produced, with GMTs of log_10_(4.60) in the low-dose group and log_10_(4.90) in the high-dose group. Following the second immunization on Day 56, antibodies against P[6]-VP8 reached peak levels, with GMTs of log_10_(5.54) in the low-dose group and log_10_(5.51) in the high-dose group. Similarly, antibodies against P[8]-VP8 reached peak levels, with GMTs of log_10_(5.48) in the low-dose group and log_10_(5.57) in the high-dose group. On Day 84, Dr14, and Dr42, titers slightly declined but remained higher than those observed after the first immunization. No significant difference in IgG levels against P[6]-VP8 and P[8]-VP8 was observed between the low- and high-dose groups. As expected, IgG was undetectable in the serum samples from the adjuvant-only group at all six time points (**Figure 5**).

### 3.6 Cellular immune responses

**Figure 6:**
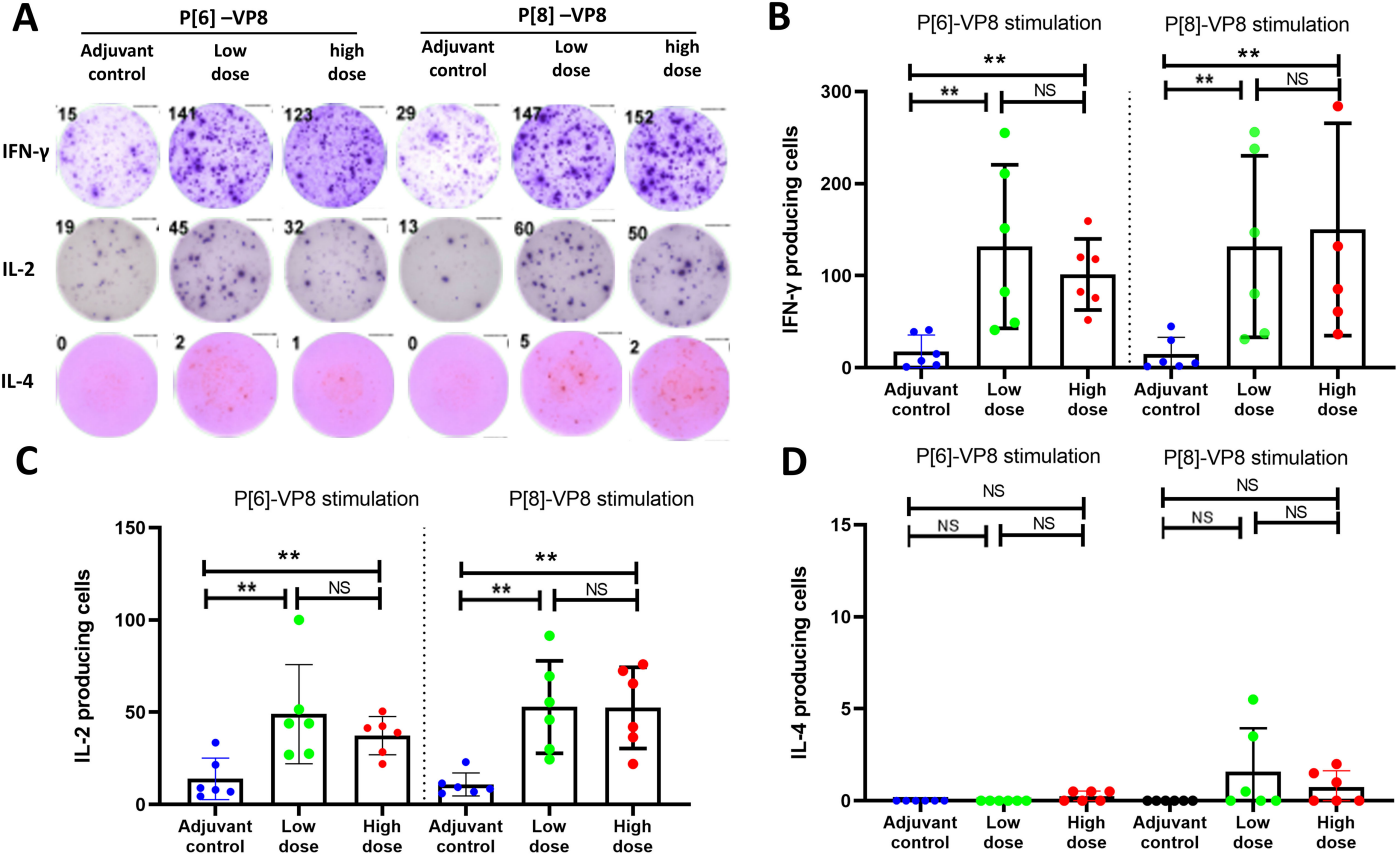
Cellular immune response to the bivalent rotavirus vaccine in cynomolgus monkeys on Dr2. **(A)** Splenocytes were plated in ELISpot wells, and IFN-γ, IL-2 and IL-4 cytokine-producing cells were detected. **(B)** IFN-γ-producing cells, **(C)** IL-2-producing cells, and **(D)** IL-4-producing cells were detected by ELISpot after stimulation with either P[6]-VP8 or P[8]-VP8. Splenocytes were collected on Dr2 following the fourth immunization. n = 6 animals/group. Comparisons were performed using one-way ANOVA. **P < 0.01 compared to the adjuvant control group.

To assess the antigen-specific cellular immune response to P[6]-VP8 and P[8]-VP8 stimulation, splenocytes were isolated from cynomolgus monkeys following four immunizations and analyzed at the end of the dosing phase (Dr2). T helper 1 (Th1)-secreted cytokines interleukin (IL)-2 and interferon-gamma (IFN-γ), along with T helper 2 (Th2)-secreted IL-4, were detected using the ELISpot assay. Both 60 μg and 120 μg doses of the bivalent subunit vaccine induced significantly higher numbers of IL-2- and IFN-γ-secreting cells compared to the adjuvant control group when stimulated with either P[6]-VP8 or P[8]-VP8 (*P* < 0.01). However, no statistically significant difference was observed between the high- and low-dose groups (**Figures 6A, B**). The IL-4 ELISpot assay showed negligible antigen-specific IL-4 responses in both groups after stimulation with P[6]-VP8 and P[8]-VP8 antigens, respectively (**Figure 6C**). These findings suggested that the vaccine may induce a cellular immune response characterized by elevated levels of IL-2 and IFN-γ secretion, demonstrated that the vaccine is effective in inducing antigen-specific cellular immune responses. The cellular immune responses to P[6]-VP8 and P[8]-VP8 proteins on Day 56 were comparable to those observed on Day 84 (**Supplementary Figure 1**).

### 3.7 Gross necropsy and histopathological examination

Post-mortem analysis revealed no significant differences in organ weights or organ-to-body weight ratios of major organs among the different groups. The only exception was a statistically significant increase in spleen weight observed in high-dose female monkeys compared to the adjuvant control group at the end of the recovery phase (*P* < 0.05, **Supplementary Table 4**). However, the spleen-to-body weight ratio did not differ significantly (*P* > 0.05), and no abnormalities were observed upon gross or microscopic examination of the spleen. Therefore, the isolated change in spleen weight was considered to be of no biological significance.

**Figure 7:**
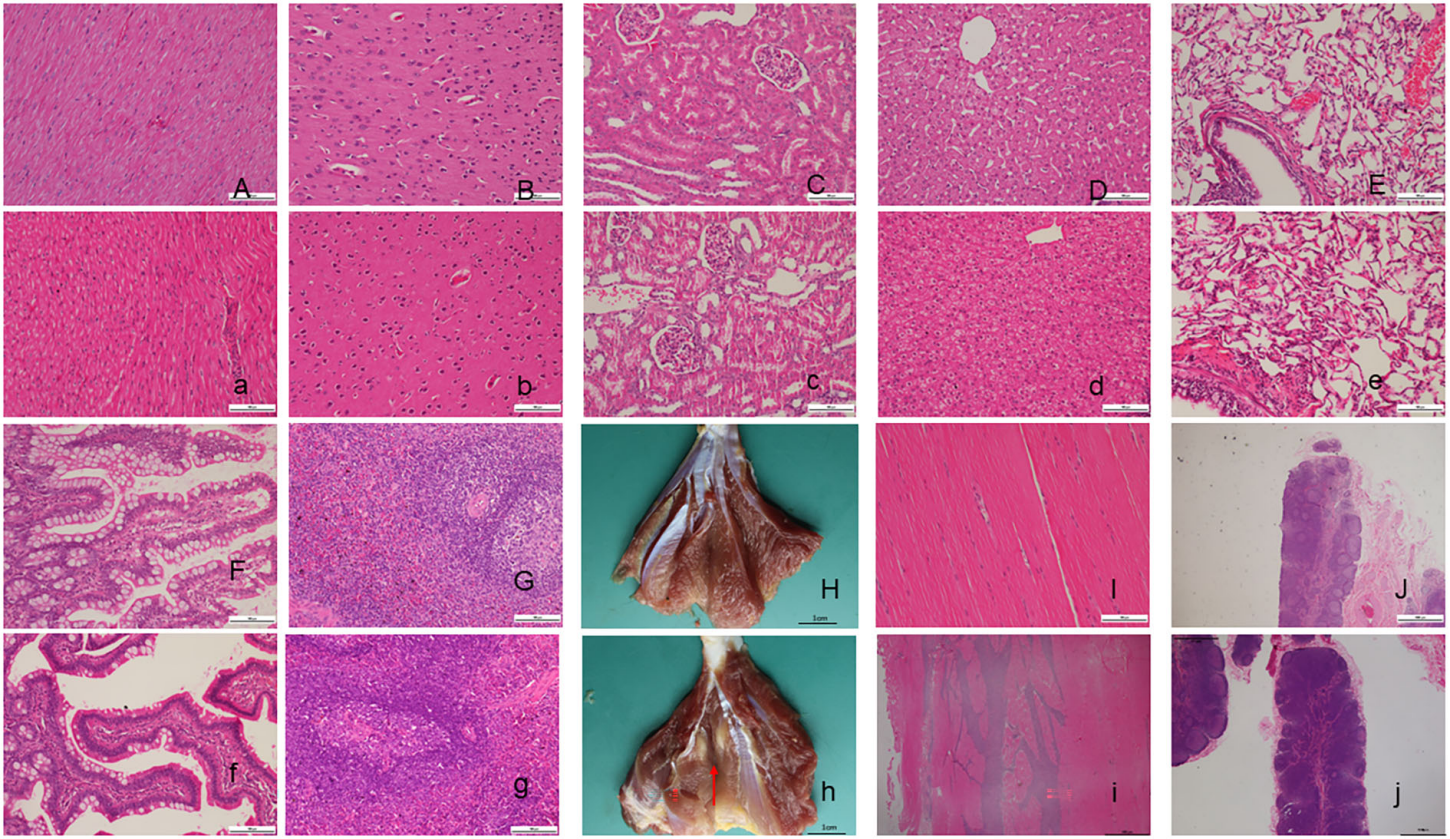
Histopathological analysis of major organs in cynomolgus monkeys following repeated bivalent vaccine administration. No significant histopathological abnormalities were observed in the heart **(A, a)**, brain **(B, b)**, kidney **(C, c)**, liver **(D, d)**, lung **(E, e)**, intestine **(F, f)**, or spleen **(G, g)** between the control group **(A–G)** and the high-dose vaccine group **(a–g)**. Representative gross and histological images of the injection site muscle and inguinal lymph nodes are shown for the control group **(H, I, J)** and the high-dose group **(h, i, j)**. The arrow indicates yellowish patchy deposits at the injection site **(h)**. Hematoxylin and eosin staining: **(A–i)**, ×200; **(J–j)**, ×20.

No vaccine-related histopathological changes were observed in the major organs examined (**Figures 7A–g**). At necropsy, yellowish patches or streaks, consistent with adjuvant deposition, were noted within the interstitium of muscle fibers in the adjuvant and vaccine groups (**Figures 7H–h**). Histological examination of the injection site revealed focal basophilic adjuvant deposits, accompanied by the infiltration of phagocytes, lymphocytes, plasma cells, and granulocytes. Mild interstitial edema and congestion were also present. The inflammatory changes were distributed along the muscle fiber spaces; however, no degeneration or necrosis of the muscle fibers was observed (**Figures 7I–i**). The alterations in the adjuvant and vaccine groups were essentially similar and characterized as a subacute inflammatory response, likely related to the injection of aluminum-containing adjuvants. Following the 6-week recovery period, the degree of inflammation showed a partial of recovery compared to that at the end of the dosing phase. At the end of the dosing phase, mild follicular hyperplasia and lymph sinus histiocytosis were observed in the inguinal (draining) lymph nodes of all animals in the adjuvant and vaccine groups (6/6). These changes persisted throughout the recovery phase and were attributed to adjuvant−induced immune activation, rather than to the vaccine antigen itself (**Figures 7J–j**).

## 4 Discussion

Chimeric virus-like particles (cVLPs) serve as a versatile vaccine engineering platform for presenting heterologous antigens, thereby facilitating the development of prophylactic or therapeutic vaccines against diverse pathogens—including viruses, bacteria, and parasites like *Plasmodium*—as well as cancers. The successful global approval of RTS, S/AS01 and R21/Matrix-M—malaria vaccines developed by fusing the *Plasmodium falciparum* circumsporozoite protein antigen to the hepatitis B surface antigen (HBsAg) VLP carrier—demonstrates the feasibility and safety profile of the cVLP technological approach. Vaccines based on a variety of other VLP platforms are under development, including those based on hepatitis B core antigen (HBcAg), bacteriophages, human papillomavirus (HPV) L1 protein, and influenza virus components. However, it is crucial to emphasize that safety data established for one specific VLP platform cannot be directly extrapolated to other structurally distinct platforms. The norovirus P particle, the subject of this study, differs fundamentally from the HBsAg VLP in key physicochemical and biological properties, such as molecular architecture, assembly mechanism, surface charge, and antigen presentation mode. Consequently, the safety characteristics of the norovirus P particle-based vaccine require independent and systematic evaluation.

This study represents the first systematic preclinical safety assessment of a bivalent chimeric rotavirus candidate vaccine (PP-P[6]/P[8]-VP8 Mix) based on a norovirus P particle platform in cynomolgus macaques. No systemic toxicological effects were observed in any experimental group. Local reactions were primarily characterized by adjuvant deposition and mild to moderate inflammatory cell infiltration at the injection site, which were observed in the adjuvant control, low-dose, and high-dose groups but not in the blank control (saline) group, indicating that these reactions were mainly attributable to the aluminum-containing adjuvant. The results of this preclinical safety evaluation in cynomolgus macaques support the advancement of this norovirus P particle-based bivalent rotavirus candidate vaccine into clinical trials. Furthermore, the safety profile established in this study provides a reference for other vaccines utilizing the norovirus P particle platform.

Currently, most preclinical vaccine toxicology studies are conducted in healthy adult animals. However, the primary target population for rotavirus infection and vaccination consists of infants and young children. Age critically shapes vaccine-induced immunity: early-life adaptive immunity differs functionally from adults, with more naïve/regulatory T cells, impaired germinal centers, reduced somatic hypermutation, and fewer long-lived plasma cells (28). These age-dependent differences are vaccine-specific: MF59-adjuvanted influenza and hepatitis B vaccines elicit stronger responses in younger children (29, 30). while mRNA-1273 induces weaker CD8+ T-cell responses in younger children (31). Thus, age-related responses cannot be generalized and require empirical evaluation per vaccine. Anatomically and physiologically, infants and young children differ significantly from adults, extending well beyond mere differences in body size. Their immune systems are immature, leading to distinct responses to pathogens and vaccines compared to those of adults (32–35). Consequently, adult animal models may inadequately predict the immunogenicity and safety profiles of vaccines in infants and young children with developing immune systems, and may fail to detect potential impacts on growth and development. The rotavirus vaccine is clinically indicated for infants and young children, aged 6 weeks to 71 months—a developmental stage comparable to that of juvenile cynomolgus macaques under 2 years of age. Therefore, this study employed juvenile animals to more accurately simulate the immunological status of infants and young children, and to specifically evaluate potential adverse effects of the vaccine on growth and development in a pediatric context. Notably, repeated administration of the bivalent vaccine did not significantly affect growth, bone development, or growth hormone levels in the juvenile monkeys (**Supplementary Tables 5**-**7**).

Serum IgG and IgA serve as critical indicators for evaluating the immune response to rotavirus vaccines. Notably, IgA plays a particularly important role not only in systemic neutralization but also as a precursor to secretory IgA, which is essential for establishing mucosal immunity at the primary site of rotavirus infection (36, 37). This study evaluated the humoral immune response induced by the bivalent vaccine in cynomolgus macaques. The vaccination schedule consisted of four dosages administered on days 0, 28, 56 and 84. High titers of P[6]-VP8- and P[8]-VP8-specific IgG antibodies were detectable as early as day 28 after the first dose (i.e., prior to the second dose), and the antibody response persisted until 42 days after the fourth dose (i.e., day 126 post-first dose), indicating durable immunogenicity. Following completion of both the two-dose and three-dose vaccination regimens, all cynomolgus macaques exhibited significantly elevated titers of P[6]-VP8 and P[8]-VP8 specific IgA antibodies. These results demonstrate that the bivalent vaccine elicits a rapid, durable, and potent serum antibody response in cynomolgus macaques. The vaccine-induced response constitutes a key component of both systemic and mucosal immunity against rotavirus infection.

A limitation of our study is the lack of neutralization antibody data for serotype P[6] rotavirus. Due to the unavailability of P[6] rotavirus strains (1076 strain, G2P[6]) for neutralization assays, we were unable to perform immunogenicity tests against the P[6] chimeric antigen. This absence of data restricts our ability to fully evaluate the vaccine’s cross-neutralization potential against P[6] strains. To partially address this limitation, we conducted neutralization antibody tests for the P[4] virus strain (DS-1 strain). The high titers of neutralizing antibodies against the P[4] virus strain provide evidence supporting the vaccine’s cross- neutralization efficacy. Neutralization studies against P[6] rotavirus strains will be conducted in future work to address this remaining gap in the neutralization profile. Although Secretory IgA (sIgA) in the intestinal mucosa is considered the most direct measure of mucosal immunity against rotavirus, serum anti-rotavirus IgA has been widely accepted as a practical surrogate marker of vaccine immunogenicity and a correlate of protection in rotavirus vaccine trials, particularly in large-scale studies where collection of intestinal samples is challenging (38–40). As stated in the WHO position paper on rotavirus vaccines (2021), serum anti-rotavirus IgA antibody responses have been used as a measure of immunogenicity of all live attenuated rotavirus vaccines evaluated (Organization, 2021). As an enteric virus, rotavirus primarily infects mature epithelial cells lining the villi of the small intestine. The intestinal IgA reflects mucosal immune activation following vaccination. Therefore, its assessment should be conducted in future studies.

The VP8 protein is relatively small (approximately 220 amino acids) and exhibits poor immunogenicity as a monomeric antigen. To overcome this limitation, we utilized the norovirus P particle as a multivalent nanoparticle platform to display the VP8 antigen. As previously demonstrated by Xia et al. (14), the P particle platform dramatically enhances the immunogenicity of fused antigens. Therefore, a VP8-alone control group was not included in this study, as the monomeric VP8 antigen is known to be poorly immunogenic. Nevertheless, we acknowledge that the empty P particle control should be included in future studies for systematic safety assessment of the P particle platform.

Currently licensed live attenuated oral rotavirus vaccines, such as Rotarix and RotaTeq, offer the advantage of directly delivering the vaccine to the intestinal tract, where it replicates and effectively induces robust mucosal immunity, particularly secretory IgA (sIgA), serving as the first line of defense against rotavirus infection (41, 42). These live attenuated oral rotavirus vaccines are associated with a low risk of intussusception and their efficacy is reduced in low- and middle-income countries, partly due to interference from maternal antibodies and concomitant enteric infections (5). In contrast, the recombinant protein vaccine candidate is administered parenterally and lacks the ability to replicate in the host, thereby theoretically eliminating the risk of vaccine-associated intussusception. Unlike live oral vaccines, our candidate vaccine primarily induces systemic humoral and cellular immunity, leading to stronger serum antibody responses and a favorable safety profile. They also offer several potential advantages, including suitability for immunocompromised children, and ease of large-scale production and so on (43–45). The advantages and disadvantages of our vaccine relative to other vaccines merit further investigation in subsequent clinical studies.

Based on the observation that the 60 µg and 120 µg doses elicited comparable humoral and cellular immune responses in cynomolgus monkeys, indicating that increasing the antigen dose from 60 µg to 120 µg did not further enhance immunogenicity. The 60 µg dose is recommended for future clinical studies as it achieves optimal immunogenicity while minimizing antigen exposure.

## 5 Conclusion

The candidate bivalent rotavirus vaccine demonstrated robust immunoreactivity and safety in cynomolgus macaques. The observed humoral and cellular response activities, along with the ability to neutralize viruses, suggest its promising potential for human use. These findings collectively indicate that the candidate vaccine exhibits favorable immunogenicity and safety profiles, thereby supporting its advancement to clinical studies.

## Data Availability

The raw data supporting the conclusions of this article will be made available by the authors, without undue reservation.

## References

[1] Troeger CE Arndt MB Aalruz H Abdoun M Abdullahi A Abebe M . Quantifying the fatal and non-fatal burden of disease associated with child growth failure 2000–2023: a systematic analysis from the Global Burden of Disease Study 2023. Lancet Child Adolesc Health. (2026) 10:22–38. doi: 10.1016/S2352-4642(25)00303-7 41344792 PMC12674951

[2] Troeger C Khalil IA Rao PC Cao S Blacker BF Ahmed T . Rotavirus vaccination and the global burden of rotavirus diarrhea among children younger than 5 years. JAMA Pediatr. (2018) 172:958–65. doi: 10.1001/jamapediatrics.2018.1960 30105384 PMC6233802

[3] Du Y Chen C Zhang X Yan D Jiang D Liu X . Global burden and trends of rotavirus infection-associated deaths from 1990 to 2019: an observational trend study. Virol J. (2022) 19:166. doi: 10.1186/s12985-022-01898-9 36266651 PMC9585833

[4] Velasquez DE Parashar U Jiang B . Decreased performance of live attenuated, oral rotavirus vaccines in low-income settings: causes and contributing factors. Expert Rev Vaccines. (2018) 17:145–61. doi: 10.1080/14760584.2018.1418665 29252042 PMC8826512

[5] Mir-Hosseinian M Behnezhad F Hosseini-Fakhr SS Kachooei A Eftekhari M Jalilvand S . Understanding the challenges of oral live attenuated rotavirus vaccines performance in low- and middle-income countries: host, pathogen, and environmental determinants. J Med Virol. (2025) 97:e70468. doi: 10.1002/jmv.70468 40586704

[6] Karla S-W Hanna B Nicholas H Femi P Nigel C . Vaccines for preventing rotavirus diarrhoea: vaccines in use. Cochrane Database Syst Rev. (2019) 2019:CD008521. doi: 10.1002/14651858.CD008521.pub5 30912133 PMC6434239

[7] Qinjian Z Shaowei L Hai Y Ningshao X Yorgo M . Virus-like particle-based human vaccines: quality assessment based on structural and functional properties. Trends Biotechnol. (2013) 31:654–63. doi: 10.1016/j.tibtech.2013.09.002 24125746

[8] Milad K Hong L Ebenezer T . Virus-like particle vaccines and platforms for vaccine development. Viruses. (2023) 15:1109. doi: 10.3390/v15051109 37243195 PMC10223759

[9] Tan M Xia M Huang P Wang L Zhong W Mcneal M . Norovirus P particle as a platform for antigen presentation. Proc Vaccinol. (2011) 4:19–26. doi: 10.1016/j.provac.2011.07.004 38826717

[10] Tan M Jiang X . Norovirus capsid protein-derived nanoparticles and polymers as versatile platforms for antigen presentation and vaccine development. Pharmaceutics. (2019) 11:472. doi: 10.3390/pharmaceutics11090472 31547456 PMC6781506

[11] Tan M Huang P Xia M Fang PA Zhong W Mcneal M . Norovirus P particle, a novel platform for vaccine development and antibody production. J Virol. (2011) 85:753–64. doi: 10.1128/jvi.01835-10 21068235 PMC3020015

[12] Blazevic V Malm M Arinobu D Lappalainen S Vesikari T . Rotavirus capsid VP6 protein acts as an adjuvant *in vivo* for norovirus virus-like particles in a combination vaccine. Hum Vaccin Immunother. (2016) 12:740–8. doi: 10.1080/21645515.2015.1099772 26467630 PMC4964741

[13] Xia M Huang P Jiang X Tan M . A nanoparticle-based trivalent vaccine targeting the glycan binding VP8* domains of rotaviruses. Viruses. (2021) 13:72. doi: 10.3390/v13010072 33419150 PMC7825513

[14] Xia M Tan M Wei C Zhong W Wang L Mcneal M . A candidate dual vaccine against influenza and noroviruses. Vaccine. (2011) 29:7670–7. doi: 10.1016/j.vaccine.2011.07.139 21839795 PMC3190067

[15] Tan M Jiang X . Norovirus P particle: a subviral nanoparticle for vaccine development against norovirus, rotavirus and influenza virus. Nanomedicine (Lond). (2012) 7:889–97. doi: 10.2217/nnm.12.62 22734641 PMC3514417

[16] Gong X Yin H Shi Y He X Yu Y Guan S . Evaluation of the immunogenicity and protective effects of a trivalent chimeric norovirus P particle immunogen displaying influenza HA2 from subtypes H1, H3 and B. Emerg Microbes Infect. (2016) 5:e51. doi: 10.1038/emi.2016.51 27222326 PMC4893548

[17] Zang Y Bi J Du D Liu X Zhang Y Su W . Development of a norovirus P particle platform for eliciting neutralizing antibody responses to the membrane proximal external region of human immunodeficiency virus type 1 envelope. Protein Pept Lett. (2014) 21:1230–9. doi: 10.2174/0929866521666140616120955 24939661

[18] Yu Y Fu L Shi Y Guan S Yang L Gong X . Elicitation of HIV-1 neutralizing antibodies by presentation of 4E10 and 10E8 epitopes on norovirus P particles. Immunol Lett. (2015) 168:271–8. doi: 10.1016/j.imlet.2015.10.003 26455781

[19] Fu L Li Y Hu Y Yu B Zhang H Wu J . Norovirus P particle: an excellent vaccine platform for antibody production against Alzheimer's disease. Immunol Lett. (2015) 168:22–30. doi: 10.1016/j.imlet.2015.09.002 26349054

[20] Li Y Fu L Hu Y Jin H Zheng Y Yin Y . Establishment of a novel method without sequence modification for developing NoV P particle-based chimeric vaccines. Protein Expr Purif. (2016) 121:73–80. doi: 10.1016/j.pep.2016.01.003 26773744

[21] Fix AD Harro C Mcneal M Dally L Flores J Robertson G . Safety and immunogenicity of a parenterally administered rotavirus VP8 subunit vaccine in healthy adults. Vaccine. (2015) 33:3766–72. doi: 10.1016/j.vaccine.2015.05.024 26065919

[22] Shalini S Michael A K Dan W Feng L . The role and implication of rotavirus VP8∗ in viral infection and vaccine development. Virology. (2025) 609:115603. doi: 10.1016/j.virol.2025.110563 40378555

[23] Tan M Jiang X . The p domain of norovirus capsid protein forms a subviral particle that binds to histo-blood group antigen receptors. J Virol. (2005) 79:14017–30. doi: 10.1128/jvi.79.22.14017-14030.2005 16254337 PMC1280206

[24] Tan M Fang P Chachiyo T Xia M Huang P Fang Z . Noroviral P particle: structure, function and applications in virus-host interaction. Virology. (2008) 382:115–23. doi: 10.1016/j.virol.2008.08.047 18926552 PMC3508508

[25] Fang H Tan M Xia M Wang L Jiang X . Norovirus P particle efficiently elicits innate, humoral and cellular immunity. PloS One. (2013) 8:e63269. doi: 10.1371/journal.pone.0063269 23638188 PMC3639243

[26] Smith JS Yager PA Baer GM . A rapid reproducible test for determining rabies neutralizing antibody. Bull World Health Organ. (1973) 48:535–41. PMC2482941 4544144

[27] Benavides JA Velasco-Villa A Godino LC Satheshkumar PS Nino R Rojas-Paniagua E . Abortive vampire bat rabies infections in Peruvian peridomestic livestock. PloS NeglTrop Dis. (2020) 14:e0008194. doi: 10.1371/journal.pntd.0008194 32598388 PMC7351222

[28] Semmes EC Chen J-L Goswami R Burt TD Permar SR Fouda GG . Understanding early-life adaptive immunity to guide interventions for pediatric health. Front Immunol. (2021) 11:595297. doi: 10.3389/fimmu.2020.595297 33552052 PMC7858666

[29] Ota MO Vekemans J Schlegel-Haueter SE Fielding K Whittle H Lambert PH . Hepatitis B immunisation induces higher antibody and memory Th2 responses in new-borns than in adults. Vaccine. (2004) 22:511–9. doi: 10.1016/j.vaccine.2003.07.020 14670334

[30] Vesikari T Knuf M Wutzler P Karvonen A Kieninger-Baum D Schmitt HJ . Oil-in-water emulsion adjuvant with influenza vaccine in young children. N Engl J Med. (2011) 365:1406–16. doi: 10.1056/NEJMoa1010331 21995388

[31] Rostad CA Campbell JD Paulsen GC Ghamloush SS Xu W Zheng L . Evaluation of cellular immune responses after mRNA-1273 vaccination in children 6 months to 11 years of age. J Infect Dis. (2025) 231:e945-e955. doi: 10.1093/infdis/jiaf144 40119775 PMC12128075

[32] Martinez DR Permar SR Fouda GG . Contrasting adult and infant immune responses to HIV infection and vaccination. Clin Vaccine Immunol. (2016) 23:84–94. doi: 10.1128/cvi.00565-15 26656117 PMC4744916

[33] Verhoeven D Perry S . Differential mucosal IL-10-induced immunoregulation of innate immune responses occurs in influenza infected infants/toddlers and adults. Immunol Cell Biol. (2017) 95:252–60. doi: 10.1038/icb.2016.91 27629065

[34] Myneni VD Mcclain-Caldwell I Martin D Vitale-Cross L Marko K Firriolo JM . Mesenchymal stromal cells from infants with simple polydactyly modulate immune responses more efficiently than adult mesenchymal stromal cells. Cytotherapy. (2019) 21:148–61. doi: 10.1016/j.jcyt.2018.11.008 30595353 PMC6435420

[35] Sarfas C White AD Sibley L Morrison AL Gullick J Lawrence S . Characterization of the infant immune system and the influence and immunogenicity of BCG vaccination in infant and adult rhesus macaques. Front Immunol. (2021) 12:754589. doi: 10.3389/fimmu.2021.754589 34707617 PMC8542880

[36] Patel M Glass RI Jiang B Santosham M Lopman B Parashar U . A systematic review of anti-rotavirus serum IgA antibody titer as a potential correlate of rotavirus vaccine efficacy. J Infect Dis. (2013) 208:284–94. doi: 10.1093/infdis/jit166 23596320

[37] Seikrit C Pabst O . The immune landscape of IgA induction in the gut. Semin Immunopathology. (2021) 43:627–37. doi: 10.1007/s00281-021-00879-4 34379174 PMC8551147

[38] Grimwood K Lund JC Coulson BS Hudson IL Bishop RF Barnes GL . Comparison of serum and mucosal antibody responses following severe acute rotavirus gastroenteritis in young children. J Clin Microbiol. (1988) 26:732–8. doi: 10.1128/jcm.26.4.732-738.1988 2835391 PMC266431

[39] Cheuvart B Neuzil KM Steele AD Cunliffe N Madhi SA Karkada N . Association of serum anti-rotavirus immunoglobulin A antibody seropositivity and protection against severe rotavirus gastroenteritis: analysis of clinical trials of human rotavirus vaccine. Hum Vaccin Immunother. (2014) 10:505–11. doi: 10.4161/hv.27097 24240068 PMC4185910

[40] Baker JM Tate JE Leon J Haber MJ Pitzer VE Lopman BA . Postvaccination serum antirotavirus immunoglobulin A as a correlate of protection against rotavirus gastroenteritis across settings. J Infect Dis. (2020) 222:309–18. doi: 10.1093/infdis/jiaa068 32060525 PMC7323497

[41] Corthésy B Benureau Y Perrier C Fourgeux C Parez N Greenberg H . Rotavirus anti-VP6 secretory immunoglobulin A contributes to protection via intracellular neutralization but not via immune exclusion. J Virol. (2006) 80:10692–9. doi: 10.1128/jvi.00927-06 16956954 PMC1641769

[42] Blutt SE Miller AD Salmon SL Metzger DW Conner ME . IgA is important for clearance and critical for protection from rotavirus infection. Mucosal Immunol. (2012) 5:712–9. doi: 10.1038/mi.2012.51 22739233 PMC3461240

[43] World Health Organization . Rotavirus vaccines: WHO position paper — July 2021. Wkly Epidemiol Rec. (2021) 96(28):301–20.

[44] World Health Organization Diarrhoeal disease. World Health Organization. In: World health organization (2024). Geneva: World Health Organization. Available online at: https://www.who.int/news-room/fact-sheets/detail/diarrhoeal-disease. [Online] (Accessed May 31, 2026).

[45] Moore SM Yager M . The Rapid Fluorescent Focus Inhibition Test. In: Rupprecht C Nagarajan T , eds. Current Laboratory Techniques in Rabies Diagnosis, Research, and Prevention. Amsterdam: Elsevier (2015) 199–215. doi: 10.1016/B978-0-12-801919-1.00017-8

